# Study of the Structure and Antimicrobial Activity of Ca-Deficient Ceramics on Chlorhexidine Nanoclay Substrate

**DOI:** 10.3390/ma12182996

**Published:** 2019-09-16

**Authors:** Lenka Pazourková, Magda Reli, Marianna Hundáková, Erich Pazdziora, Daniela Predoi, Gražyna Simha Martynková, Khalid Lafdi

**Affiliations:** 1 IT4Innovations Centre of Excellence, VŠB–Technical University of Ostrava, 17.listopadu 15/2172, 708 33 Ostrava–Poruba, Czech Republic; grazyna.simha@vsb.cz; 2 Nanotechnology Centre, VŠB–Technical University of Ostrava, 17.listopadu 15/2172, 708 33 Ostrava–Poruba, Czech Republic; MSamlikova@seznam.cz (M.R.); marianna.hundakova@vsb.cz (M.H.); 3 Regional Materials Science and Technology Centre, VŠB–Technical University of Ostrava, 17.listopadu 15/2172, 708 33 Ostrava–Poruba, Czech Republic; 4 Institute of Public Health Ostrava, Centre of Clinical Laboratories, Partyzánské náměstí 7, 702 00 Ostrava, Czech Republic; erich.pazdziora@zuova.cz; 5 National Institute of Materials Physics, P.O. Box MG 07, 077125 Magurele, Romania; dpredoi@gmail.com; 6 University of Dayton, 300 College Park, Dayton, OH 45469, USA; klafdi1@udayton.edu

**Keywords:** Ca-deficient hydroxyapatite, chlorhexidine, organoclay, antimicrobial effect

## Abstract

Novel biomedical composites, based on organically modified vermiculite and montmorillonite with deposited Ca-deficient hydroxyapatite (CDH), were prepared. The monoionic sodium forms of vermiculite and montmorillonite were intercalated with chlorhexidine diacetate (CA). The surfaces of organoclays were used for the precipitation of Ca-deficient hydroxyapatite. The composites with Ca-deficient hydroxyapatite showed very good antibacterial effects, similar to the antimicrobial activity of pure organoclay samples. Better antibacterial activity was shown in the organically modified montmorillonite sample with Ca-deficient hydroxyapatite compared with the vermiculite composite, but, in the case of *Staphylococcus aureus*, both composites showed the same minimum inhibitory concentration (MIC) value. The antimicrobial effect of composites against bacteria and fungi increased with the time of exposure. The structural characterization of all the prepared materials, performed using X-ray diffraction and FT infrared spectroscopy analysis, detected no changes in the original clay or CDH during the intercalation or precipitation process, therefore we expect the strength of the compounds to be in the original power.

## 1. Introduction

Oral diseases are a worldwide issue. The exterior of teeth is composed of enamel, which has remarkable hardness and resistance. The composition of enamel consists of needle-like apatite crystals, which are bundled in parallel ordered prisms to ensure the unique mechanical strength and biological protection. Even though teeth are well-protected, bacteria can often damage the enamel and cause tooth decay. These days fillings with artificial materials are being used to fix tooth problems. Amalgam, metal alloys, ceramics, or composite resins are used for conventional treatment to repair damaged enamel, but this treatment often leads to secondary tooth decay at the interface between the tooth and foreign materials. One of the promising ways to suppress secondary tooth decay is to add hydroxyapatite, the model compound of enamel, into the filling [1]. 

In recent years, calcium phosphates have been widely used as attractive materials for biological and medicinal applications. Synthetic hydroxyapatite and its modifications are used due to their similarity to and compatibility with human hard tissues (e.g., bones, enamel). Most research has mainly focused on hydroxyapatite (HAp) preparation, but fewer studies have targeted calcium-deficient hydroxyapatite, Ca_10−*x*_(HPO4)*_x_*(PO_4_)_6−*x*_(OH)_2−*x*_ (0 < *x* <1) (CDH). CDH is a very promising material for biomedical utilization due to its resemblance to human hard tissue [2,3,4].

One possible way to deliver Ca-deficient hydroxyapatite into the desired area is in the form of a nanocomposite, where the base material of the nanocomposite is clay mineral. Clay minerals are already widely used in pharmaceutical application as either active agents (having therapeutic properties) or excipients [5]. Clay minerals have several important properties, such as surface reactivity (cation exchange, swelling, and absorption), solubility, large specific surface area, non-toxicity for human, etc. 

Ambre et al. (2011) used organo-modified montmorillonite (MMT) as precursor for the in situ preparation of hydroxyapatite. Hydroxyapatite created in the MMT gallery exhibits differences in its lattice structure compared to ex situ prepared HAp. Afterwards, the composite of MMT/in situ HAp was added into the chitosan/polygalacturonic acid composite films. This composite may be used as bone biomaterials [6].

The goal of the paper was to study the antimicrobial effect of the complex hybrid material possible interactions, which aims to be an optimal biomaterial for use in prosthetics and bone reconstruction. This study focused on the preparation of Ca-deficient hydroxyapatite nanocomposite with antibacterial properties. Two different clay minerals (vermiculite and montmorillonite) were used as supporting materials. To ensure the antibacterial properties of this novel composite, chlorhexidine was intercalated into the clay minerals interlayer. To the best of our knowledge this composite has not been prepared before. 

## 2. Experimental

### 2.1. Materials

The natural clay mineral vermiculite (VER) from Santa Luzia, Brazil and montmorillonite (MMT) from Ivančice, Czech Republic, purchased from Grena, a.s., Veselí nad Lužnicí, Czech Republic, were selected as starting materials. As received mineral powders were ground in a planetary ball mill for 20 min and then sieved using a 45 μm mesh sieve. The particle size fractions less than 45 μm were used for the experiment. The crystalo-chemical formula of both clay minerals was determined based on the result of the elemental chemical analysis for the half unit cell as:

VER: (Si_3.13_Al_0.87_) (Mg_2.53_Fe${}_{0.45}^{3+}$Al_0.02_) O_10_(OH)_2_ (Mg_0.19_K_0.01_Ca_0.02_), and

MMT: (Si_7.96_Al_0.04_) (Al_2.52_Fe^3+^_0.54_Mg_0.90_Ti_0.04_) O_20_(OH)_4_ (Ca_0.24_K_0.06_Na_0.09_Mg_0.10_).

The cation exchange capacities (CEC) were 140 cmol(+)/kg for VER and 105 cmol(+)/kg for MMT, and were established via the Cd^2+^ exchange-detection method using spectrometers. The chemicals used for modification of the MMT and VER samples were sodium chloride (NaCl, Vitrum VWR, Co., Stříbrná Skalice, Czech Republic), chlorhexidine diacetate (C_22_H_30_N_10_Cl_2_.2C_2_H_4_O_2_, Sigma Aldrich, Prague, Czech Republic), and ethanol (CH_3_CH_2_OH, Vitrum VWR, Co., Stříbrná Skalice, Czech Republic) as a solvent.

As precursors for the preparation of Ca-deficient hydroxyapatite (CDH), sodium phosphate dibasic dodecahydrate (Na_2_HPO_4_∙12H_2_O, Vitrum VWR, Co., Stříbrná Skalice, Czech Republic) and calcium chloride dihydrate (CaCl_2_∙2 H_2_O, Vitrum VWR, Co., Stříbrná Skalice, Czech Republic) were used.

### 2.2. Modifications and Preparation of Samples

The natural VER and MMT were converted into their monoionic Na form (NaVER and NaMMT, respectively) by cation exchange. MMT and VER were mixed with an aqueous solution of NaCl (1.0 mmol∙dm^−3^) and heated at 70 °C for 2 h. The NaCl solution was replaced for a fresh solution after 2 h of intercalation, for a total of 3 times to achieve maximal saturation of Na^+^ ions. The clay suspension was then centrifugated (at 3000 rpm for 15 min), washed with demineralized water several times until it was free of chloride ions, and left to dry at 80 °C for 24 h. Dried samples of NaVER and NaMMT were intercalated with ethanolic solution of chlorhexidine diacetate (CA). The concentration of CA for this modification was calculated according to the cation exchange capacity of VER (1 × CEC). The batch process of intercalation persisted for 6 h at 75 °C, keeping the suspension free of any motion. After centrifugation and drying at 80 °C overnight, the organovermiculite (VCA) and organomontmorillonite (MCA) were obtained. 

A total of 100 mL of CaCl_2_ solution (120 mmol∙dm^−3^ in deionized water) was slowly added to the continuously mixed solution of Na_2_HPO_4_ (72 mmol∙dm^−3^ in deionized water) which contained 1 g of MCA or VCA. pH of resulting solution was adjusted to 7.45 by 1 mol∙dm^−3^ HCl. The precipitate was let to sediment for 24 h. After this period the supernatant was decanted and the precipitate was dried at 70 °C. The samples were abbreviated as MCAH (for MCA + CDH) and VCAH (for VCA+ CDH).

### 2.3. Analytical Methods

X-ray diffraction (XRD) patterns were obtained using the diffractometer Ultima IV (RIGAKU, Tokyo, Japan) (Bragg–Brentano arrangement, CuKα radiation, reflection mode, NiK_β_ filter, 40 kV, 40 mA, ambient atmosphere, fine powder sample placed at non-diffracting holder, angle range 2–60° 2θ, scan speed 2°/min). The international centre for diffraction data (ICDD) database powder diffraction file (PDF) -4+ 2019 for phase analysis was used. The diffraction data were drawn using Origin 9.1. software. 

The images of samples were acquired using scanning electron microscopy (SEM) Philips XL-30 (Eindhoven, Netherland) tungsten filament, equipped with EDAX energy dispersive spectrometer (EDS), using a secondary electrons (SE) detector, and at working conditions of 25 kV acceleration voltage. The samples were coated using gold/palladium to improve conduction (layer thickness is several nm). Elemental analysis using EDS point measurement was done for each sample at 5 measurement points.

The KBr method was used to obtain IR spectra of samples using a Nexus 470 Fourier-transform (FTIR) spectrometer (ThermoNicolet, Waltham, MA, USA) equipped with a KBr beam splitter, Globar IR source, and deuterated-triglycine sulfate (DTGS) detector. The 128 scans were obtained with a resolution of 4 cm^−1^ in the range of 400–4000 cm^−1^ for each spectrum.

The Brunauer–Emmett–Teller (BET) specific surface area (SSA) was obtained using the porosimeter Surfer (Thermo Fisher Scientific, Waltham, MA, USA) by adsorption/desorption of N_2_ at the temperature of liquid nitrogen. The samples were degased at 70 °C for 4 h. The samples were dried before analysis for 24 h at 80 °C.

### 2.4. Antibacterial Test

The minimum inhibitory concentration (MIC) (the lowest concentration of sample that completely inhibits bacterial growth) was determined as the antibacterial activity of the prepared samples. The micro-titration plate with 96 hollows was used for the dilution and cultivation. The 10% (w/v) samples in water dispersion were prepared into the first set of hollows on the plate. These dispersions were further diluted to obtain concentration of 3.33%, 1.11%, 0.37%, 0.12%, 0.041%, and 0.014% (w/v) by a threefold diluting method in glucose stock. 

One set of hollows contained pure glucose stock as a reference test. The hollows were filled with 1 µL glucose suspensions of *Escherichia coli* CCM 3954 (1.1 × 10^9^ cfu mL^−1^), *Enterococcus faecalis* CCM 4224 (1 × 10^9^ cfu mL^−1^), *Pseudomonas aeruginosa* CCM 1960 (1.1 × 10^9^ cfu mL^−1^), *Staphylococcus aureus* CCM 3953 (1 × 10^9^ cfu mL^−1^), and *Candida albicans* ATC 90028 (1.1 × 10^9^ cfu mL^−1^), provided by the Czech collection of microorganisms (CCM). Bacterial suspensions were transferred from each hollow to 100 µL of the fresh glucose stock after an elapse of 30, 60, 90, 120, 180, 240 and 300 min, and then over 5 days at 24 h intervals, and bacteria were incubated at 37 °C for 24 h and 48 h [7].

## 3. Results and Discussions

### 3.1. X-ray Diffraction Analysis

**Figure 1:**
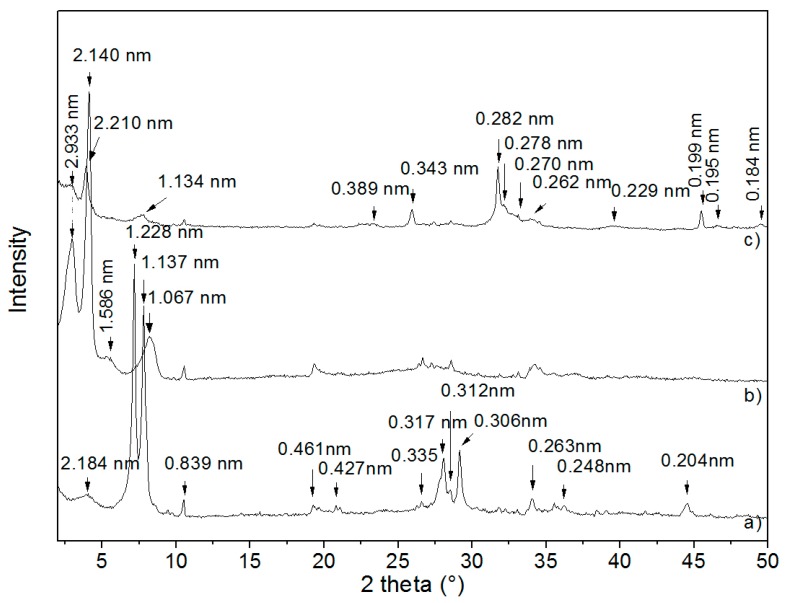
XRD patterns of VER samples (**a**) NaVER; (**b**) VCA; (**c**) VCAH at CuKα radiation.

The XRD patterns of the monoionic NaVER sample, the organovermiculite VCA sample, and the sample of composite VCAH are shown in Figure 1.

The XRD pattern of NaVER (Figure 1a) shows a basal reflection with an interlayer distance *d* = 1.228 nm, corresponding to the Na^+^ cations as the interlayer material with one layer of water molecules. The other reflections, with *d* = 2.184 nm and d = 1.137 nm, may be described as the interstratified layered structure with different hydration states [8,9]. The other reflections for the NaVER correspond to *d*-values of 0.461 nm, 0.427 nm, 0.335 nm, 0.306 nm, 0.263 nm, 0.248 nm, and 0.204 nm. Reflections with *d*-values 0.839 nm and 0.312 nm belong to the admixture phase of amphibole mineral tremolite (ICDD, PDF card no. 00-013-0437) and value *d* = 0.317 nm was ascribed to the admixture of rutile (ICDD, PDF card no. 01-071-4809) [10]. After NaVER intercalation with CA (Figure 1b), the interlayer space expanded to *d* = 2.933 nm, *d* = 2.140 nm, *d* = 1.586 nm, and *d* = 1.067 nm. These new series of reflections confirmed intercalation of CA into the VER interlayer [11]. 

After precipitation of CDH, the relative intensities of the mentioned reflections were rapidly reduced in the VCAH (Figure 1c). Interlayer distances shifted to *d* = 2.981 nm, *d* = 2.210 nm, and *d* = 1.134 nm. These changes may signify the release of a small amount of CA from the VER interlayer and reorganization of CA molecules in the VER interlayer [11,12,13]. 

Reflections of VCAH (Figure 1c) with *d*-values 0.389 nm, 0.343 nm, 0.282 nm, 0.278 nm, 0.271 nm, 0.262 nm, 0.229 nm, 0.199 nm, 0.195 nm, and 0.184 nm correspond to the values for the hexagonal hydroxyapatite structure from PDF card no. 01-075-9526. Thus, the XRD pattern of VCAH confirmed that CDH is distributed on the VER surface, which is in agreement with the findings from the SEM.

**Figure 2:**
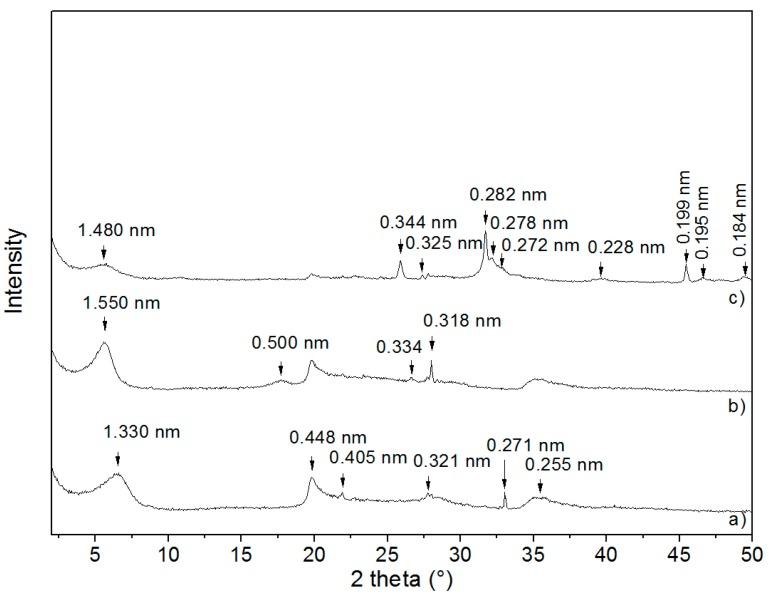
XRD patterns of MMT samples (**a**) NaMMT, (**b**) MCA, (**c**) MCAH CuKα radiation.

The XRD pattern of NaMMT (Figure 2a) shows a basal reflection with interlayer distance *d* = 1.33 nm. The other reflections for the NaMMT correspond to *d*-values of 0.448 nm, 0.405 nm, 0.321 nm, 0.271 nm, and 0.255 nm. 

After NaMMT intercalation with CA (Figure 2b), the interlayer space expanded from *d* = 1.33 nm to *d* = 1.55 nm, which indicates the intercalation of CA into the MMT interlayer. 

After precipitation of CDH, the relative intensity of this reflection was reduced and the interlayer distance shifted to *d* = 1.48 nm in MCAH (Figure 2c). Similarly as with VCAH, these changes may signify a small release of CA from the MMT interlayer and the small reorganization of CA molecules in the VER interlayer [12,13]. Nevertheless, as well as in sample VCAH, the reflections of CDH (PDF card no. 01-075-9526) were present in the XRD pattern of MCAH (Figure 2c), confirming CDH on the MMT surface in agreement with SEM-edax data and images.

The CDH crystallite sizes (*L_c_*) were calculated using Scherrer’s equation [14]. The *L_c_* was calculated based on (002) reflection (about 25.9° 2θ) since this reflection was not influenced by the VER or MMT phase. The CDH crystallite size was 30.16 nm for VCAH and 31.79 nm for MCAH. 

### 3.2. FTIR Spectroscopy

**Figure 3:**
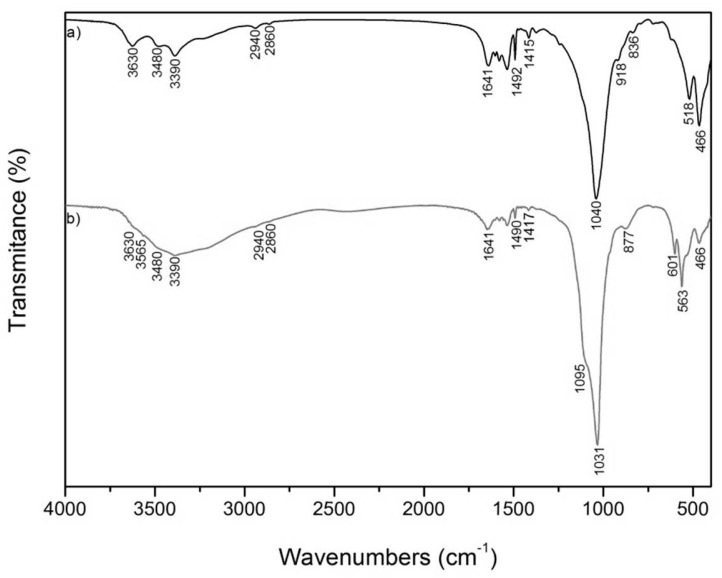
FTIR spectra of: (**a**) MCA and (**b**) MCAH.

The IR spectra of the organically-modified clay MCA and the mixture of that modified clay with CDH, marked as MCAH, are shown in Figure 3.

The IR spectrum of the MCA sample (Figure 3a) shows absorptions at 3630 and 3480 cm^−1^ in the OH stretching region of MCA. These bands were attributed to the structural OH groups and the H–O–H stretching vibration of water molecules [15]. The very intense band at 1040 cm^−1^ belongs to Si–O stretching vibration. Absorptions at 918 cm^−1^ belong to AlAlOH, and at 836 cm^−1^ to AlMgOH deformation vibration [16]. The absorption band near 800 cm^−1^ was assigned to the Si-O vibration of silica [17]. The Si–O–Al and Si–O–Si deformation vibrations were observed at 518 cm^−1^ and 466 cm^−1^ in the MCA spectrum. Characteristic bands at 3390 cm^−1^, 2940 cm^−1^, and 2860 cm^−1^ correspond to the asymmetric NH stretching bands and asymmetric and symmetric C–H stretching bands of CA [18]. The bands that occurred in the 1590–1492 cm^−1^ interval were due to the NH bending vibration of secondary amine and imine groups. The stretching vibration of the imine group appears at 1641 cm^−1^ [18,19]. Absorption at 1415 cm^−1^ belongs to the C=C stretching vibration of an aromatic ring [20].

The IR spectrum of MCAH (Figure 3b) was a little bit changed in comparison with the IR spectrum of MCA and it showed the identification of characteristic bands corresponding to CDH. Characteristic bands at 563, 601, 1031and 1095 cm^−1^, were attributed to the phosphate group (PO_4_^3−^) and the band at 3565 cm^−1^ belonged to structural OH^−^ group [21]. There were no significant shifts after preparation of CDH on the organically modified MMT, so it is expected that there are no chemical interactions between CDH and MMT.

**Figure 4:**
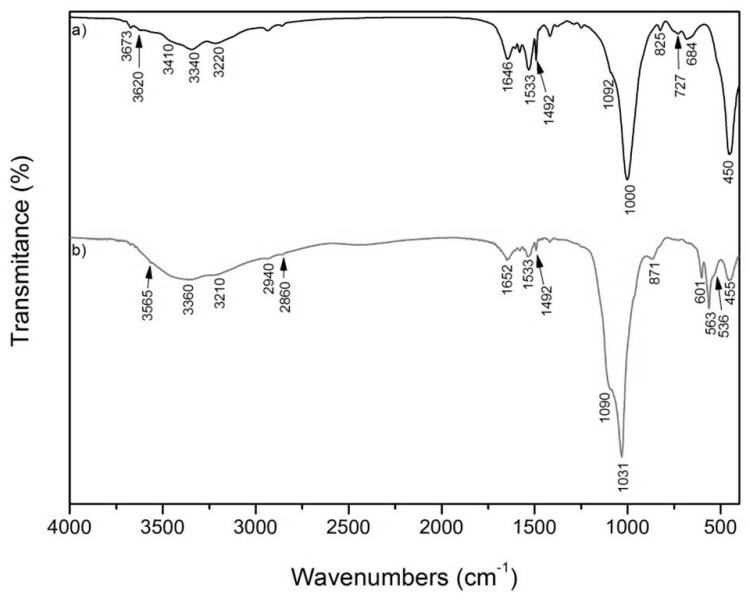
FTIR spectra of: (**a**) VCA and (**b**) VCAH.

The IR spectrum of VCA is shown in Figure 4. There is a band in the OH stretching region at 3673 cm^−1^ attributed to the Mg_3_OH unit, which belongs with the absorption at 684 cm^−1^ to the OH bending vibration. These bands suggest a trioctahedral character of VER [15]. Absorptions at 3620 cm^−1^ correspond to the Fe_2_OH unit. Absorption observed at 3410 cm^−1^ belongs to the OH stretching vibration of adsorbed water, and the band around 1646 cm^−1^ corresponds to the OH bending vibration of adsorbed water. The bands at 1000 and 450 cm^−1^ belong to Si–O stretching and Si–O bending vibrations. 

The presence of CA (Figure 4a,b) was confirmed by absorption of C–H stretching of methylene group at 2940 and 2860 cm^−1^, C–H stretching of chlorophenyl group at 3220 cm^−1^, C–H out-of-plane bending with respect to the benzene ring at 825 cm^−1^, C–N stretching of -NR_2_ group at 727 cm^−1^, C=N stretching of N_2_-C=N– group around 1646 cm^−1^, and finally –CH_2_-bending of methylene group at 1492 cm^−1^ [22].

Figure 4b shows the IR spectrum of the Ca-deficient hydroxyapatite composite with organovermiculite (VCAH). The presence of CA was confirmed by absorptions at 3360, 3210, 2940, 2860, 1652, and 1533 cm^−1^ [18]. The characteristic bands of internal phosphate (PO_4_^3−^) were observed in the spectrum of VCAH. The presence of two characteristic bands around 563 and 601 cm^−1^ correspond to ν_4_ (OPO) bending mode. The doublet absorption at 1031–1090 cm^−1^ was assigned to ν_3_ (PO) antisymmetric stretching mode. These bands indicate the characteristic molecular structures of the polyhedrons of PO_4_^3−^ in the apatite lattice [23]. Also, in the VCAH, there were no significant shifts after preparation of CDH on the organically modified VER, so it is expected that there are no chemical interactions between CDH and VER [24].

**Figure 5:**
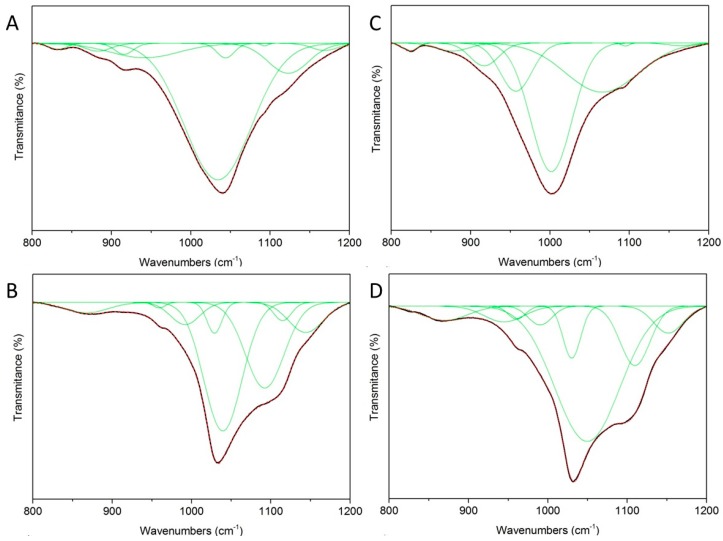
Deconvoluted FTIR spectra of (**a**) MCA, (**b**) VCA, (**c**) MCAH and (**d**) VCAH (experimental curve: black, calculated curve: red dash, sub-bands: green).

The peak fitting analysis of the IR spectra in the region of 800–1200 cm^−1^ was performed for proper identification of the process that takes place during preparation of the composite. Figure 5 shows the experimental and calculated contours overlaid along with the individual sub-bands, as determined by a curve fitting analysis. All fitting bands with appropriate domains are listed in Table 1. The MCA sample contained bands of MMT (883, 941, 1034, and 1122 cm^−1^) and CA (832, 915, 1043, and 1168 cm^−1^). The band corresponding to 1093 cm^−1^ should be assigned to both MMT (Si-O) and CA (C–Cl stretching vibration of aromatic halogen compounds).

Nine components were needed for the satisfactory fit of MCA. Eight components were needed for satisfactory fit of the MCAH sample. In this sample, bands of MMT (867, 991, 1114 and 1144 cm^−1^), CDH (959, 1028, 1039 and 1092 cm^−1^), and CA (1092 cm^−1^) were identified. The band at 1092 cm^−1^ was attributed to CDH or CA.

The VCA sample needed 8 components connected with VER (977, 917, 956, 1001 and 1064 cm^−1^) and CA (1164 cm^−1^) for a satisfactory fit. The remaining bands, 823 and 1094 cm^−1^, can be attributed to VER or CA. Nanocomposite VCAH reached a successful fit with 8 components, and also identified bands only of VER (869, 990 and 1109 cm^−1^) and CDH (944, 960, 1049 and 1151 cm^−1^). Bands of CA were not identified.

The 8 components were necessary for the satisfactory fit of all samples, except the MCA sample. The MCA sample needed 9 components to fit. The absence of components corresponding to the CA should be caused by low-intensity bands of CA and high-intensity bands of VER and CDH at that region.

### 3.3. Antimicrobial Test

Antibacterial and antifungal tests were performed against two gram-positive (*E. faecalis*, *S. aureus*) and two of gram-negative (*E. coli*, *P. aeruginosa*) bacterial strains, and one yeast strain (*C. albicans*). Table 2 shows chosen results (after 0.5, 2, 4, 24 and 120 h) for the MIC values, which were necessary for inhibition of microbial growth.

The samples for NaMMT and NaVER showed no antibacterial effect. After intercalation of CA into the interlayer space of NaMMT and NaVER, the values of MIC declined. The best results were obtained after a longer time of exposure (120 h). So, we can conclude that these materials can be used as antibacterial compounds with a long-lasting effect. Even for very resistant bacteria *P. aeruginosa*, very positive results have been obtained. The higher antibacterial activity, in this case, was proved for the MCA sample. Prepared biocomposite samples, MCAH and VCAH, displayed very good antibacterial activity against all tested bacteria. The composite MCAH reached the antibacterial activity of MCA against *S. aureus*, *E. coli*, and *P. aeruginosa*. In the case of *E. faecalis*, MCAH nearly reached the MIC value of MCA. The VCAH composite showed the same MIC values with VCA against *S. aureus* and *P. aeruginosa*. VCAH exhibited smaller MIC (after 4 and 24 h) values *against P. aeruginosa* in comparison with VCA, but after 120 h the MIC values were the same for both samples. In all of the tests, the sample MCAH showed better antibacterial activity than VCAH. The biggest appreciable difference in the antibacterial effect of VCAH and MCAH was in test for *P. aeruginosa*—sample VCAH showed very poor activity compared to MCAH.

The antifungal activity against yeast *C. albicans* was also very promising. The Na forms of both clay minerals did not show antifungal activity as was expected. On the other hand, organo-modified clays exhibited good antifungal activity. The VCA showed, after 120 h of exposure, that the MIC value was 0.041% (w/v). The MCA showed a gradual decrease of the MIC value, and after 4 h of contact time, the MIC value was 0.014 % (w/v), which did not change until the end of the test. Composite MCAH showed a little bit higher MIC values, but still had a very good antifungal effect. The higher MIC value of MCAH than that of MCA might be caused by the presence of CDH, which is a biocompatible material. A very similar situation occurred in case of the VER composite. VER modified by CA showed good antifungal activity. Nevertheless, the VCA showed activity against *C. albicans* up to 2 h of contact with the yeast.

### 3.4. Scanning Electron Microscopy

**Figure 6:**
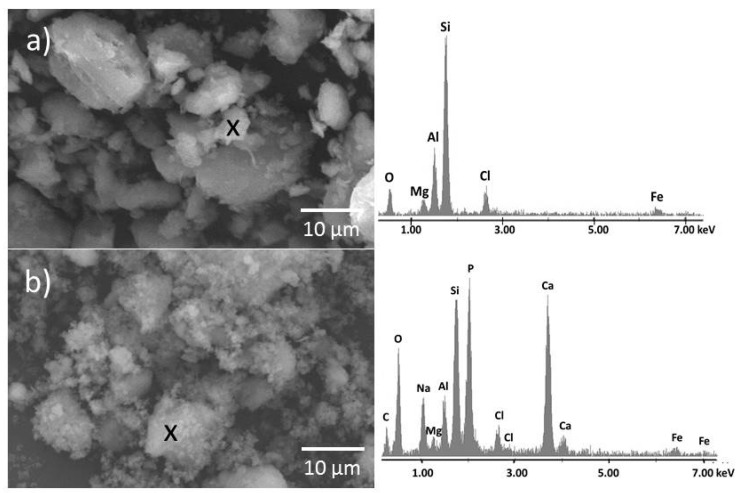
SEM image of (**a**) MCA and (**b**) MCAH with adequate representative EDS analysis.

Figure 6a shows the organo-modified MMT at 2000× magnification in a back-scattered electron detector. The MCA exhibited particles of different size. The surface of the MCA particles is smooth. There were relatively large individual particles and also very small individual particles, which probably do not create large agglomerates/aggregates. The EDS analysis of the small particles showed the presence of elements that correspond to MMT (O, Na, Mg, Al, Si) and also CA (Cl, O).

Figure 6b shows the SEM micrograph of composite MCAH at 2000× magnification in a back-scattered electron detector. Preparation of CDH on MCA surface led to the creation of a large amount of agglomerates/aggregates, consisting of very small particles and large particles. The surfaces of the large MCA particles are visibly covered by a CDH “film” and also by very small CDH particles. The CDH particles are visibly attached on the MMT surface. The EDS analysis confirmed the presence of elements characteristic for MMT (O, Na, Mg, Al, Si), CA (C, Cl) and, of course, for CDH (Ca, P).

**Figure 7:**
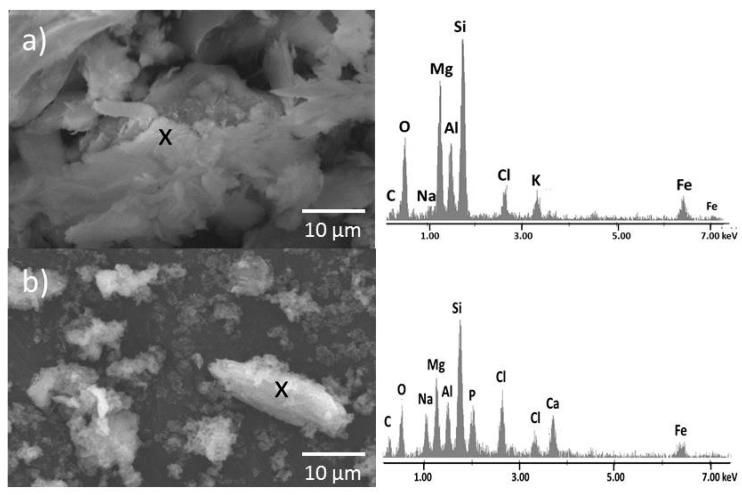
SEM image of (**a**) VCA and (**b**) VCAH with adequate representative EDS analysis.

A very similar situation, as in case of organo-MMT, occurred in organo-VER samples. In Figure 7a particles of the VCA sample are visible, detected by the back-scattered electron detector at 2000× magnification. There are visible platelets of VER, which exhibit a relatively smooth surface. The small VER particles create clusters. The sample, of course, contained larger VER particles. The EDS analysis showed elements characteristic for both components—VER (O, Na, Mg, Al, Si, K, Ca) and CA (C, Cl).

Figure 7b shows the photo of composite VCAH at 2000× magnification in back-scattered electron detection. The VER particles became coarse because of a covering by CDH particles. The CDH covers the VER surface either as a CDH “film” or the surface is covered by clusters of very small CDH particles. The small VCAH particles make clusters. The EDS analysis confirmed the presence of elements characteristic for VER (O, Na, Mg, Al, Si, K), CA (C, Cl) and CDH (Ca, P).

The coarse surface of clay minerals after CDH preparation could have affected little bit worse antibacterial activity, but, on the other hand, the rough surface can facilitate the attachment of bone cells.

### 3.5. Specific Surface Area Measurement

The modification of VER and MMT by chlorhexidine diacetate led to a decrease of SSA (VER → VCA: from 24.25 m^2^/g to 7.04 m^2^/g; MMT → MCA: from 77.86 m^2^/g to 15.84 m^2^/g), which is caused by the covering of the clay mineral surface by chlorhexidine diacetate molecules. The SSA increased after preparation of CDH on the organically-modified clay mineral. The SSA of VCAH was higher (39.54 m^2^/g) than in case of VER (24.25 m^2^/g), and the SSA of MCAH (61.19 m^2^/g) nearly reached the SSA of MMT (77.86 m^2^/g).

## 4. Conclusions

Novel antibacterial nanocomposites, which combined organoclays and hydroxyapatite, were prepared. From a structural point of view, XRD and FTIR confirmed the presence of the stable antibacterial agent chlorhexidine, along with hydroxyapatite. The structure and interlayer space of the supporting clays were not changed during the modification process (confirmed with XRD analysis)—no shifts of position of basal reflections of individual nanocomposite components were observed. Only MCAH showed small shifts of MMT basal reflection, which was probably caused by a partial release of CA from the MMT interlayer space. This should be caused by rinsing the MCA in water during CDH preparation. FTIR analysis confirmed that no chemical interactions between CDH and organomodified clay minerals had occurred. The antibacterial activity of the prepared samples is very promising for application in prosthetics or orthopedic surgery, where infection may take place. Both composites showed antibacterial activity of organically modified clays using two gram-positive (*E. faecalis*, *S. aureus*) and two of gram-negative (*E. coli*, *P. aeruginosa*). The better results for antibacterial activity generally showed sample with montmorillonite, rather than vermiculite, due to the higher amount of CA on montmorillonite.

SEM observation showed clay minerals were covered by CDH particles. On the clay mineral surface, the CDH creates either clusters of very small particles or covers the surface like a CDH “film”. The smooth clay mineral surface became coarse after preparation with CDH particles. The SSA measurement confirmed enlargement of the specific surface area after CDH precipitation. This could facilitate improved options for attachment of bone cells.

## Figures and Tables

**Table 1:** FTIR bands associated with the functional groups present in all the samples.

MCA (cm^−1^)	MCAH (cm^−1^)	VCA (cm^−1^)	VCAH (cm^−1^)	Assignments
-	-	823	-	C–H vibration deformation of methylene group [25]
832	-	-	-	C–H out-of-plane vibration of 1,4-substitued aromatic ring [26]
-	867	877	869	AlO_4_, Al–O stretching vibration [27]
883	-	-	-	AlFe^3+^OH [28]
915	-	917	-	Al–OH–Al [29]
941	-	-	944	Si–O symmetric [30]
-	-	956	-	Si–O stretching [31]
-	959	-	960	ν_1_ PO_4_^3−^ [32]
-	991	1001	990	Si-O-Si [30]
-	1028	-	1030	ν_3_ PO_4_^3−^ [33]
1034	-	-	-	Si–O stretching vibration [34]
-	1039	-	-	ν_3_ PO_4_^3−^ [33]
1043	-	-	-	Aromatic amine CA [35]
-	-	-	1049	Aromatic amine [35]
-	-	1064	-	ν_asym_Si–O [36]
1093	1092	1094	-	Si–O, C–Cl stretching vibration of halogen compounds [37]
-	-	-	1109	Asymmetric Si–O–Si [38]
-	1114	-	-	Si–O stretching [38]
1122	-	-	-	Si–O–Si [39]
-	1144	-	1151	Symmetric Si–O [40]
1168	-	1164	-	C–OH stretching [41]

**Table 2:** The MICs (%, w/v) of prepared samples against the different bacterial strains.

**Sample**	***Enterococcus Faecalis*** **MIC (%, w/v)**					***Staphylococcus Aureus*** **MIC** **(%, w/v)**				
	0.5 h	2 h	4 h	24 h	120 h	0.5 h	2 h	4 h	24 h	120 h
NaMMT	>10	>10	>10	>10	>10	>10	>10	>10	>10	>10
MCA	3.33	3.33	3.33	0.014	0.014	0.37	0.041	0.014	0.014	0.014
MCAH	3.33	3.33	3.33	1.11	0.12	1.11	1.11	1.11	0.014	0.014
NaVER	>10	>10	>10	>10	>10	>10	>10	>10	>10	10
VCA	10	3.33	10	0.014	0.014	0.37	0.12	0.014	0.014	0.014
VCAH	10	10	10	10	0.37	3.33	3.33	3.33	0.014	0.014
	***Escherichia Coli*** **MIC (%, w/v)**					***Pseudomonas Aeruginosa*** **MIC** **(%, w/v)**				
	0.5 h	2 h	4 h	24 h	120 h	0.5 h	2 h	4 h	24 h	120 h
NaMMT	>10	>10	>10	>10	>10	>10	>10	>10	>10	>10
MCA	1.11	1.11	1.11	0.014	0.014	3.33	3.33	3.33	1.11	1.11
MCAH	3.33	1.11	1.11	0.12	0.014	10	1.11	3.33	1.11	1.11
NaVER	>10	>10	>10	>10	>10	>10	>10	>10	>10	>10
VCA	3.33	1.11	1.11	0.014	0.014	10	10	10	10	10
VCAH	10	3.33	3.33	0.12	0.37	10	10	3.33	3.33	10
	***Candida Albicans*** **MIC (%, w/v)**					-				
	0.5 h	2 h	4 h	24 h	120 h					
NaMMT	>10	>10	>10	>10	>10					
MCA	0.12	0.041	0.014	0.014	0.014					
MCAH	10	1.11	0.12	1.11	0.37					
NaVER	>10	>10	>10	>10	>10					
VCA	>10	0.12	0.041	0.014	0.041					
VCAH	>10	0.37	1.11	1.11	0.37					
